# Evaluation of C‐terminal Agrin Fragment as a marker of muscle wasting in patients after acute stroke during early rehabilitation

**DOI:** 10.1002/jcsm.12068

**Published:** 2015-10-27

**Authors:** Nadja Scherbakov, Michael Knops, Nicole Ebner, Miroslava Valentova, Anja Sandek, Ulrike Grittner, Pius Dahinden, Stefan Hettwer, Jörg C Schefold, Stephan von Haehling, Stefan D. Anker, Michael Joebges, Wolfram Doehner

**Affiliations:** ^1^ Center for Stroke Research CSBCharite Universitätsmedizin BerlinGermany; ^2^ German Centre for Cardiovascular Research (DZHK), partner site BerlinGermany; ^3^ Innovative Clinical Trials, Department of Cardiology and PneumologyUniversity Medicine GöttingenGermany; ^4^ 1st Department of Internal MedicineComenius UniversityBratislavaSlovak Republic; ^5^ Neurotune AGWagistrasse 27aSchlierenSwitzerland; ^6^ Department of Intensive Care MedicineInselspital, University Hospital of BernSwitzerland; ^7^ Department of NeurologyBrandenburgklinik BernauGermany; ^8^ Department of CardiologyCharite Universitätsmedizin BerlinGermany

**Keywords:** Stroke, Muscle wasting, Post‐stroke rehabilitation, C‐terminal Agrin Fragment, Physical performance, Skeletal muscle mass

## Abstract

**Background:**

C‐terminal Agrin Fragment (CAF) has been proposed as a novel biomarker for sarcopenia originating from the degeneration of the neuromuscular junctions. In patients with stroke muscle wasting is a common observation that predicts functional outcome. We aimed to evaluate agrin sub‐fragment CAF22 as a marker of decreased muscle mass and physical performance in the early phase after acute stroke.

**Methods:**

Patients with acute ischaemic or haemorrhagic stroke (n = 123, mean age 70 ± 11 y, body mass index BMI 27.0 ± 4.9 kg/m^2^) admitted to inpatient rehabilitation were studied in comparison to 26 healthy controls of similar age and BMI. Functional assessments were performed at begin (23 ± 17 days post stroke) and at the end of the structured rehabilitation programme (49 ± 18 days post stroke) that included physical assessment, maximum hand grip strength, Rivermead motor assessment, and Barthel index. Body composition was assessed by bioelectrical impedance analysis (BIA). Serum levels of CAF22 were measured by ELISA.

**Results:**

CAF22 levels were elevated in stroke patients at admission (134.3 ± 52.3 pM) and showed incomplete recovery until discharge (118.2 ± 42.7 pM) compared to healthy controls (95.7 ± 31.8 pM, p < 0.001). Simple regression analyses revealed an association between CAF22 levels and parameters of physical performance, hand grip strength, and phase angle, a BIA derived measure of the muscle cellular integrity. Improvement of the handgrip strength of the paretic arm during rehabilitation was independently related to the recovery of CAF22 serum levels only in those patients who showed increased lean mass during the rehabilitation.

**Conclusions:**

CAF22 serum profiles showed a dynamic elevation and recovery in the subacute phase after acute stroke. Further studies are needed to explore the potential of CAF22 as a serum marker to monitor the muscle status in patients after stroke.

## Introduction

Skeletal muscle wasting has been frequently observed after stroke.^1^ Already within 4 h after cerebral damage an initial reduction of motoneurons in the musculature of paretic limb is observed^2^ that persists in the chronic phase after stroke.^3^ Loss of muscle innervation leads to muscular weakness, inactivity, and immobilization and results in muscle atrophy. Within the first week after stroke muscle weakness occurs also in the non‐paretic limb.^4^ Decline of muscle mass has been observed in stroke patients within first three weeks after hemiparetic stroke.^5^ Further, patients who are not able to relearn walking within 2 months after stroke revealed similar lean mass reduction in paretic and non‐paretic leg.^6^ A combination of mechanisms, including immobilization, disuse, inflammation, metabolic, and neurovegetative imbalance after stroke, results frequently in muscle wasting and may progress to the stroke‐related sarcopenia.^1^, ^7^ The presence of stroke‐specific sarcopenia has been proposed from experimental^8^ and clinical data.^9^, ^10^

Progressive degradation of muscle mass was termed as ‘sarcopenia’ and was originally observed in relationship to aging.^11^ The prevalence of sarcopenia is about 5 to 10% in persons over 65 years of age.^12^ Numerous factors such as malnutrition,^13^ immobilization and disuse,^14^ hormonal imbalance etc. are discussed in the multifactorial aetiology of sarcopenia.^15^, ^16^, ^17^, ^18^ In aging loss of motoneurons has been proposed as pathogenic and contributing to the developing of sarcopenia.^19^, ^20^

**Figure 1:**
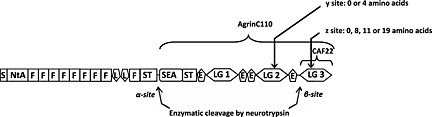
Structure and cleavage sites of agrin.

Recently, C‐terminal Agrin Fragment (CAF) has been proposed as a potential marker for sarcopenia caused by degeneration of the neuromuscular junctions (NMJs) in elderly.^21^ Agrin is a heparin sulphate proteoglycan with a molecular weight of 225 kDa, which is considered as a key organizer of postsynaptic differentiation at NMJs.^22^, ^23^, ^24^ Proper clustering of acetylcholine receptors (AChR) at post‐synaptic basal lamina depends on agrin‐mediated signalling.^22^, ^25^ Proteolytic cleavage of agrin by neuronal protease neurotrypsin at NMJs triggers inactivation and destabilization of the NMJ with subsequent muscle degradation. A sarcopenic phenotype has been observed in transgenic mouse with neurotrypsin overexpression.^26^ In human plasma two stable and bio‐inactive circulating fragments of agrin—AgrinC110 (cleavage at α‐site) and CAF22 (cleavage at β‐site) were identified *(Figure* 1).^21^ It has been shown that elevated CAF22 plasma levels may indicate muscle wasting in pre‐frail community‐dwelling older adults because of degeneration of the NMJ.^27^ The reduction of CAF22 levels after 12 week power training supports CAF22 as a marker of muscle wasting and the development of sarcopenia. In contrast, a study evaluating an effect of resistance training in older adults revealed elevation of CAF levels following 6‐weeks of training.^28^

The aim of the present study was to evaluate agrin as a marker of muscle wasting in patients with stroke. Because physical exercise is an effective therapy to prevent muscle wasting,^29^ we investigated a cohort of stroke patients in the early post stroke rehabilitation. We hypothesized that CAF22 might be a marker of muscle status and function during recovery after paretic stroke. We evaluated CAF22 in relation to changes in muscle mass and functional recovery during early rehabilitation period.

## Patients and methods

### Study population and enrolment criteria

We studied 123 patients (age ranging from 42 to 98 years) with confirmed diagnosis of ischaemic or haemorrhagic stroke. Patients were admitted from October 2011 to August 2013 to neurological rehabilitation centre Brandenburgklinik Bernau, Germany. Clinical and functional examinations were performed at admission and at discharge. Within the early post‐stroke hospitalized rehabilitation programme all patients were on standard medical therapy according to current guideline recommendations (including antiplatelet drugs, statins, angiotensin‐converting enzyme inhibitors, and β‐blocker). Exclusion criteria for this observational study were acute and chronic inflammatory diseases, acute heart failure or myocardial infarction, liver cirrhosis, acute and chronic renal failure and dialysis, immune suppressive therapy, and history of cancer within the last 5 years. Twenty six healthy individuals of similar body mass index (BMI) and age were enrolled used as control group. The research protocol was approved by the local ethics committee, and written informed consent was obtained from all subjects.

### Assessment of functional capacity and physical examination

Functional independence was assessed using the Barthel index (BI) that addresses basic self‐care and mobility aspects with a score ranging from 0 to 100, where the lowest score indicates greater dependency.^30^ Assessment of physical status included following testing: the Rivermead motor assessment gross function subscale (RMA) that scores a range of physical activities with increasing complexity from turning over in bed to hop on the affected leg 5 times.^31^, ^32^ Arm strength was analysed using the handgrip dynamometer (Saehan Corporation, Korea). The highest of three handgrip measurements was used for analysis.

### Body composition

Body mass index (BMI) was calculated as a ratio of body weight and squared height (kg/m^2^). Body composition was assessed by bioelectrical impedance analysis (BIA) (QuadScan 4000, Bodystat Limited, UK). The principle of BIA analyses is based on measurements of whole body resistance (*R*) and reactance (*Xc*) values^33^ where *R* reflects conductivity through ionic solutions, and *Xc* reflects dielectric properties of plasma membrane measured as a phase‐shift in current flow at 50 Hz. Phase angle of the whole body (*ϕ*, arc tangent expressing a relationship between Xc and R) is understood as bioimpedance measures of cell membranes of skeletal muscle and as indicator of cellular health.^34^ BIA measurements were taken in supine position in standard condition as described previously.^35^

### Blood samples

Venous blood samples were obtained under standardized conditions after overnight fasting and after 15 min of supine resting in a quiet and air conditioned room. Samples were centrifuged at 3500 rpm for 15 min (2000x g), aliquoted and stored at −80°C until analysis. CAF22 concentrations were measured using a commercially available enzyme‐linked immunosorbent assay (ELISA) kit (NTCAF Elisa Kit; Neurotune, Schieren, Switzerland) as described previously.^36^ The coefficient of variance of the test is 12.3% maximal deviation for serum and 5.6% maximal deviation for the used calibrator in a combined intra‐ and inter‐plate comparison. For the tested samples, the deviation must have been lower than 20% in each double measurement. The accuracy of the calibrator curve was >0.98 (Rsqr), and the validated range of detection was 20 pM to 380 pM. In case of higher CFA values, the sample was diluted with PBS and remeasured until the value was within the detection range.

### Statistical analysis

All variables were tested for normal distribution using the Kolmogorov–Smirnov test. All data were presented as mean values ± standard deviation or as median [interquartile range, IQR]. Paired Student t‐test, unpaired Student t‐test, and Mann–Whitney test were used as appropriate. Chi Square test was used to assess categorical distribution between groups. The relationship between variables was analysed by linear and multiple regression analyses. A value of p < 0.05 was considered statistically significant. For statistical analysis, standard statistical software packages were used (Statview 5.0, SAS Institute, Cary, NC).

## Results

### Clinical and functional characteristics before and after rehabilitation

Functional assessments were performed at admission (23 ± 17 days post stroke) and at discharge from the rehabilitation centre (49 ± 18 days post stroke).

**Figure 2:**
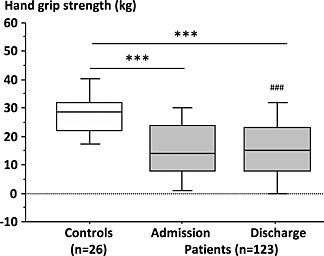
Hand grip strength of stroke patients compared to healthy controls (***p < 0.001 vs. controls; ^###^p < 0.001 vs. admission).

Baseline characteristics of study population groups are shown in *Table* 1. Patients and healthy controls were of similar age and body mass index (BMI). Fifty‐five per cent of the stroke patients revealed a 2.2% increase of the lean mass at discharge, whereas in the rest of the patients revealed a decline of the body lean mass by 2.6% (*Table* 2). Physical performance and muscle functional measures were significantly impaired after stroke compared to controls (data shown for the hand grip strength, *Figure* 2) and improved during rehabilitation as assessed by maximum hand grip strength, Barthel index, and Rivermead motor assessment (*Table* 2). Thus, patients presented better functional performance at discharge from the rehabilitation centre compared to admission.

**Table 1:** Clinical characteristics of study groups

Parameters	Controls n = 26	Patients at admission n = 123	Patients at discharge n = 123
Age, y	67 ± 8	70 ± 11	70 ± 11
Gender, f/m [m, %]	17/9 [41]	49/74 [60]*	
Stroke ischaemic, n [%] / haemorrhagic, n [%]		106 [86] / 17 [16]	
Days after stroke		23 ± 17	49 ± 18###
CAF22, pMol	95.7 ± 31.8	134.3 ± 52.3***	118.2 ± 42.7*, ###
CAF22, pMol, female	102.6 ± 30.7	140.3 ± 51.6**	119.5 ± 35.5###
CAP22, pMol, male	82.7 ± 31.2	130.2 ± 52.7**	117.3 ± 47.1*, ###
Change of total CAF22, pMol			−16.1 ± 30.4
Creatinine, mg/dL	0.79 ± 0.14	0.97 ± 0.31**	0.96 ± 0.33
Sodium, mmol/L	141.5 ± 3.3	140.1 ± 4.4	140.3 ± 2.7
Potassium, mmol/L	4.4 ± 0.4	4.4 ± 0.4	4.3 ± 0.5
Body mass index, kg/m^2^	25.6 ± 3.3	27.0 ± 4.9	26.0 ± 5.2
Lean mass, kg	46.9 ± 10.1	51.9 ± 11.7*	51.6 ± 11.3
Fat mass, kg	24.9 ± 6.9	25.0 ± 9.7	23.4 ± 8.8
Phase angle, *ϕ*	5.7 ± 1.1	5.1 ± 1.0**	5.0 ± 1.0**

* p < 0.05,

** p < 0.01,

*** p < 0.001 vs. controls;

### p < 0.001 vs. admission

**Table 2:** Parameters of body composition and physical performance at admission and at discharge from the rehabilitation centre

Parameters	At admission (n = 120)	At discharge (n = 120)
Lean mass increase, kg, n = 67	50.6 ± 11.8	51.7 ± 11.7***
Lean mass decrease, kg, n = 56	53.2 ± 11.7	51.7 ± 11.4***
Max hand grip strength paretic arm, kg	15.8 ± 11.6	16.0 ± 11.7***
Max hand grip strength nonparetic arm, kg	28.2 ± 11.1	29.6 ± 11.7***
Barthel Index score	60 ± 22	73 ± 20***
Rivermead Motor Assessment score	5.3 ± 2.0	7.2 ± 2.2***

*** p < 0.001 vs. admission

### CAF22 plasma level during rehabilitation

**Figure 3:**
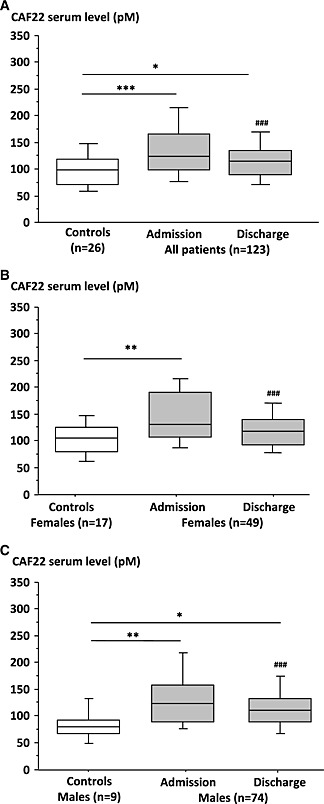
CAF22 plasma level in stroke‐patients at admission to rehabilitation centre and at discharge compared to controls: All patients (A); Female patients (B); Male patients (C) (***p < 0.001, **p < 0.01, *p < 0.05 vs. controls; ^###^p < 0.001 vs. admission).

At admission, CAF22 serum level was significantly elevated (+26%) in stroke patients compared to controls (p < 0.001, unpaired t‐test; *Figure* 3 *A*). CAF22 level declined subsequently during rehabilitation but remained 17% above the control group at discharge (p < 0.05; *Figure* 3 *A*). The mean change of CAF22 serum level between discharge and admission was expressed as a change of total CAF22 (*Table* 1). CAF22 serum levels were not significantly different in females compared to male patients (*Table* 1). However, compared to healthy controls female stroke patients showed 21% higher CAF22 level at admission (*Figure* 3 *B*), whereas between male patients and controls this difference was 56% (*Figure* 3 *C*). At discharge, CAF22 was 12% higher in female but still 41% higher in male patients compared to controls of the same gender. Thus, CAF22 level rise after stroke seems to be more pronounced in male than in female patients.

### CAF22 and functional performance

In the linear regression analyses CAF22 levels were associated with BI (r = 0.2, p < 0.022), RMA score (r = 0.2, p < 0.05), hand grip strength of the non‐paretic arm (r = 0.2, p < 0.05), age (r = 0.4, p < 0.001), and creatinine levels (r = 0.7, p < 0.001).

In multivariable regression analyses change of CAF22 serum level was independently associated with improvement of the hand grip strength of the paretic arm, but not in the non‐paretic arm in patients who showed increased muscle mass during rehabilitation (*Table* 3 *B*). However, this association was not observed in the whole study cohort (*Table* 3 *A*).

**Table 3:** A). Multiple regression analyses investigating change of hand grip strength during rehabilitation in stroke patients (n = 123)

Parameter	Coefficient	p	r
1. Change of hand grip strength of paretic arm vs.			0.286
Gender	0.059	0.579	
Age	−0.229	0.035	
Change of CAF22	−0.175	0.097	
Creatinine	−0.102	0.353	
2. Change of hand grip strength of nonparetic arm vs.			0.294
Gender	0.141	0.151	
Age	−0.157	0.120	
Change of CAF22	−0.121	0.223	
Creatinine	−0.189	0.069	

### CAF22 levels and phase angle

A strong association between CAF22 level at admission and phase angle (r = −0.351, p < 0.001) was observed in simple regression analysis. After adjustment for age, creatinine level, and gender this association remained independently significant (*Table* 4).

**Table 4:** Multiple regression analysis investigating phase angle as a parameter of cell membrane integrity in stroke patients

Parameter	Coefficient	p	r
Phase angle vs.			0.523
Gender	0.170	0.055	
Age	−0.341	0.0003	
Creatinine	0.095	0.422	
CAF22	−0.267	0.026	

## Discussion

The major finding of the study is the elevation and dynamic change of CAF22 serum levels in patients after acute stroke. CAF22 serum levels were significantly increased in the subacute phase after stroke. During rehabilitation, an incomplete return of elevated CAF22 levels was observed. CAF22 was associated with parameters of physical and functional performance and with bioelectrical impedance phase angle. Further, an improvement of hand grip strength of the paretic arm during rehabilitation was independently associated with the reduction of CAF22 serum levels in patients who showed an increase of muscle mass during rehabilitation.

We observed elevated CAF22 serum levels at admission to inpatient rehabilitation. CAF22 levels decreased during 4 weeks of a rehabilitation programme but were still higher at discharge compared to healthy subjects. Our findings are in line with previous reports showing a reduction of elevated CAF22 levels after a training programme in a cohort of elder subjects.^27^ These authors suggested CAF22 as a potential marker of age‐associated sarcopenia caused by the degeneration of the NMJ. In our study patients were admitted to the rehabilitation hospital 3 weeks after stroke. At this time point patients revealed significantly reduced handgrip strength in parallel to elevated CAF22 serum levels. In addition, our analyses have shown an independent association between reduction of CAF22 serum levels and improvement of hand grip strength of the paretic arm in patients with increased muscle mass during rehabilitation. Decreasing of CAF22 levels after 4 weeks of a physical rehabilitation programme might therefore suggest termination of the muscle wasting and reactive NMJs recovery.

Recently, CAF22 has been evaluated as a biomarker of kidney function. Indeed, agrin is expressed in the kidney and CAF22 has been related to the damage of the glomerular basement membrane.^37^, ^38^ In our study patients with renal failure as well as with dialysis were excluded. We observed a strong correlation between CAF22 and creatinine, although the reduction of the creatinine serum level at discharge was not significant in contrast to the significant reduction of the CAF22 serum levels. Thus, it is not clear to which extent CAF22 serum levels reflect the kidney function in stroke patients. Yet, a confounder for CAF22 serum levels because of kidney function could not be excluded and may be seen as a limitation of the study.

In addition, the existence of several splicing isoforms of agrin should be considered in the evaluation of CAF22 as a biomarker for sarcopenia.^22^, ^39^ Agrin function is highly regulated by alternative splicing and proteolytic processing. Splicing isoforms containing 0 or 4 amino acid inserts at the y splicing site of the LG2 domain and 0, 8, 11, or 19 (8 + 11) amino acid inserts at the z splicing site of the LG3 domain of the C‐terminus have been investigated (*Figure* 1).^23^, ^40^ The neural agrin containing 4 and 8 amino acid inserts at the y and z sites, respectively, has a high affinity to the AChR clustering, while muscle agrin and agrin found in other non‐neuronal cells lacks inserts and fails to cluster AChRs.^41^ However, splicing isoforms of agrin lacking inserts have been found in NMJs (motor neurons, skeletal muscle, and Schwann cells), in the central nervous system and peripheral tissues (lung and kidney).^23^ The ELISA assay used in the present study predominately identifies the z0 splicing isoform. Previous experiments have shown that the z0 splicing isoform is at least 10–20 folds overrepresented over the insert baring splice isoforms (Western blots and internal analyses). The appearance of the z0 splicing variant of the C‐terminal agrin fragments in blood, however, represents the activity of neurotrypsin.^42^ Further, a vast amount of z0 isoform of agrin is present on the postsynaptic side, which is also cleavable by neurotrypsin. Muscle agrin is concentrated at the nerve‐induced AChR clusters where it contributes to maturation and stabilization of the receptors.^43^, ^44^ Therefore, the postsynaptic muscle agrin is able to liberate CAF22, which may then be secreted and may appear in the blood stream. Thus, we believe that in stroke patients a significant amount of the CAF22 in serum origins from the nervous tissue or postsynaptic tissue because of denervation and degradation of the NMJs. However, optimization of the ELISA assay towards identifying of the neuronal agrin might improve the specificity and sensitivity of the results and may contribute to the establishing of CAF22 as a marker of muscle wasting caused by NMJ degeneration.

Our analyses revealed an independent association of the BIA phase angle with CAF22 levels. Phase angle has been shown in relation to the muscle mass and muscle strength; therefore phase angle represents a simple index of the integrity of the skeletal muscle cell membranes.^34^ A previous study in patients with neuromuscular diseases has shown a decline of phase angle in parallel with disease progression that was accompanied by a subsequent decline of muscle strength and quality of muscle tissue.^45^ Therefore our data suggest lower CAF22 to indicate a better cellular integrity of muscle tissue.

The present study had same limitations. As mentioned above, renal function may be a relevant confounder of CAF 22 levels. Further, BIA assessment may provide only limited information on body composition and more detailed information on tissue distribution and composition may be desirable. Previous studies comparing body composition assessment by BIA and dual‐energy X‐ray absorptiometry DEXA, or by BIA and magnet resonance imaging MRI, confirmed reliable agreement between these methods.^46^, ^47^, ^48^, ^49^, ^50^ Another study examining muscle mass assessed by BIA and MRI indicated a strong relation between muscle mass and body resistance.^49^ However, underestimation of the fat mass and over predicting of the fat‐free mass assessed by BIA has been reported.^47^ In addition, the presence of oedema may influence BIA measurements. The effect of the whole body water changes and its dependence from the sodium plasma concentrations has been discussed previously.^51^ However, in the present study patients were free of peripheral oedema and sodium plasma levels remained unchanged suggesting stable fluid balance during the observation period.

In conclusion, CAF22 serum levels were elevated in the subacute phase after acute stroke and fell during rehabilitation. Associations between CAF22 and parameters of physical performance, muscle strength, and muscle membrane integrity have been observed. In multivariable analysis recovery of increased CAF22 levels was independently associated with improved hand grip strength only in those patients who showed increasing lean tissue during rehabilitation but not in the entire cohort. The present data are promising to explore further the role CAF22 as a potential serum marker for monitoring muscle status in patients after stroke. Further studies are warranted including optimization of the analytic assay of CAF to evaluate the role of CAF22 as a serum marker of muscle wasting in stroke patients.

## Conflict of interest

NS, MK, NE, MV, AS, UG, SvH, SDA, UD, MJ, and WD: no conflict of interest. PD and SH are employed by Neurotune AG that develops the CAF biomarker.

## Acknowledgements

We thank Mrs. Anja Kresse for her excellent technical assistance with this study. The authors certify that they comply with the ethical guidelines for authorship and publishing of the Journal of Cachexia, Sarcopenia and Muscle *(von Haehling S, Morley JE, Coats AJS, Anker SD. Ethical guidelines for authorship and publishing in the Journal of Cachexia, Sarcopenia and Muscle. J Cachexia Sarcopenia Muscle. 2010; 1:7–8.)*

## References

[1] Scherbakov N , von Haehling S , Anker SD , Dirnagl U , Doehner W . Stroke induced Sarcopenia: muscle wasting and disability after stroke. Int J Cardiol 2013;170:89–94. 24231058 10.1016/j.ijcard.2013.10.031

[2] Arasaki K , Igarashi O , Ichikawa Y , Machida T , Shirozu I , Hyodo A , *et al* Reduction in the motor unit number estimate (MUNE) after cerebral infarction. J Neurol Sci 2006;250:27–32. 16904126 10.1016/j.jns.2006.06.024

[3] Li X , Shin H , Zhou P , Niu X , Liu J , Rymer WZ . Power spectral analysis of surface electromyography (EMG) at matched contraction levels of the first dorsal interosseous muscle in stroke survivors. Clin Neurophysiol 2014;125:988–994. 24268816 10.1016/j.clinph.2013.09.044

[4] Harris ML , Polkey MI , Bath PM , Moxham J . Quadriceps muscle weakness following acute hemiplegic stroke. Clin Rehabil 2001;15:274–281. 11386397 10.1191/026921501669958740

[5] Carin‐Levy G , Greig C , Young A , Lewis S , Hannan J , Mead G . Longitudinal changes in muscle strength and mass after acute stroke. Cerebrovasc Dis 2006;21:201–207. 16401884 10.1159/000090792

[6] Jørgensen L , Jacobsen BK . Changes in muscle mass, fat mass, and bone mineral content in the legs after stroke: a 1 year prospective study. Bone 2001;28:655–659. 11425655 10.1016/s8756-3282(01)00434-3

[7] Scherbakov N , Sandek A , Doehner W . Stroke‐related Sarcopenia: specific characteristics. J Am Med Dir Assoc 2015;16:272–276. 25676847 10.1016/j.jamda.2014.12.007

[8] Springer J , Schust S , Peske K , Tschirner A , Rex A , Engel O , *et al* Catabolic signaling and muscle wasting after acute ischemic stroke in mice: indication for a stroke‐specific sarcopenia. Stroke 2014;45:3675–3683. 25352483 10.1161/STROKEAHA.114.006258

[9] De Deyne PG , Hafer‐Macko CE , Ivey FM , Ryan AS , Macko RF . Muscle molecular phenotype after stroke is associated with gait speed. Muscle Nerve 2004;30:209–215. 15266637 10.1002/mus.20085

[10] Scherbakov N , Doehner W . Sarcopenia in stroke‐facts and numbers on muscle loss accounting for disability after stroke. J Cachexia Sarcopenia Muscle 2011;2:5–8. 21475676 10.1007/s13539-011-0024-8 PMC3063875

[11] Rosenberg IH . Sarcopenia: origins and clinical relevance. J Nutr 1997;127:990S–991S. 9164280 10.1093/jn/127.5.990S

[12] Morley JE , Anker SD , von Haehling S . Prevalence, incidence, and clinical impact of sarcopenia: facts, numbers, and epidemiology—update 2014. J Cachexia Sarcopenia Muscle 2014;5:253–259. 25425503 10.1007/s13539-014-0161-y PMC4248415

[13] Wakabayashi H , Sakuma K . Rehabilitation nutrition for sarcopenia with disability: a combination of both rehabilitation and nutrition care management. J Cachexia Sarcopenia Muscle 2014;5:269–277. 25223471 10.1007/s13539-014-0162-x PMC4248414

[14] Barbat‐Artigas S , Dupontgand S , Pion CH , Feiter‐Murphy Y , Aubertin‐Leheudre M . Identifying recreational physical activities associated with muscle quality in men and women aged 50 years and over. J Cachexia Sarcopenia Muscle 2014;5:221–228. 24737111 10.1007/s13539-014-0143-0 PMC4159483

[15] Muscaritoli M , Anker SD , Argilés J , Aversa Z , Bauer JM , Biolo G , *et al* Consensus definition of sarcopenia, cachexia and pre‐cachexia. Clin Nutr 2010;29:154–159. 20060626 10.1016/j.clnu.2009.12.004

[16] Fanzani A , Conraads VM , Penna F , Martinet W . Molecular and cellular mechanisms of skeletal muscle atrophy: an update. J Cachexia Sarcopenia Muscle 2012;3:163–179. 22673968 10.1007/s13539-012-0074-6 PMC3424188

[17] Palus S , von Haehling S , Springer J . Muscle wasting: an overview of recent developments in basic research. J Cachexia Sarcopenia Muscle 2014;5:193–198. 25163459 10.1007/s13539-014-0157-7 PMC4159486

[18] Henwood TR , Keogh JW , Reid N , Jordan W , Senior HE . Assessing sarcopenic prevalence and risk factors in residential aged care: methodology and feasibility. J Cachexia Sarcopenia Muscle 2014;5:229–236. 24737112 10.1007/s13539-014-0144-z PMC4159491

[19] Drey M , Grösch C , Neuwirth C , Bauer JM , Sieber CC . The Motor Unit Number Index (MUNIX) in sarcopenic patients. Exp Gerontol 2013;48:381–384. 23376626 10.1016/j.exger.2013.01.011

[20] Alchin DR . Sarcopenia: describing rather than defining a condition. J Cachexia Sarcopenia Muscle 2014;5:265–268. 25092476 10.1007/s13539-014-0156-8 PMC4248413

[21] Hettwer S , Dahinden P , Kucsera S , Farina C , Ahmed S , Fariello R , *et al* Elevated levels of a C‐terminal agrin fragment identifies a new subset of sarcopenia patients. Exp Gerontol 2013;48:69–75. 22433628 10.1016/j.exger.2012.03.002

[22] Bezakova G , Ruegg MA . New insights into the roles of agrin. Nat Rev Mol Cell Biol 2003;4:295–308. 12671652 10.1038/nrm1074

[23] Ruegg MA , Bixby JL . Agrin orchestrates synaptic differentiation at the vertebrate neuromuscular junction. Trends Neurosci 1998;21:22–27. 9464682 10.1016/s0166-2236(97)01154-5

[24] Ferraro E , Molinari F , Berghella L . Molecular control of neuromuscular junction development. J Cachexia Sarcopenia Muscle 2012;3:13–23. 22450265 10.1007/s13539-011-0041-7 PMC3302983

[25] Zong Y , Jin R . Structural mechanisms of the agrin–LRP4–MuSK signaling pathway in neuromuscular junction differentiation. Cell Mol Life Sci 2013;70:3077–3088. 23178848 10.1007/s00018-012-1209-9 PMC4627850

[26] Bütikofer L , Zurlinden A , Bolliger MF , Kunz B , Sonderegger P . Destabilization of the neuromuscular junction by proteolytic cleavage of agrin results in precocious sarcopenia. FASEB J 2011;25:4378–4393. 21885656 10.1096/fj.11-191262

[27] Drey M , Sieber CC , Bauer JM , Uter W , Dahinden P , Fariello RG , Vrijbloed JW , FiAT intervention group . C‐terminal Agrin Fragment as a potential marker for sarcopenia caused by degeneration of the neuromuscular junction. Exp Gerontol 2013;48:76–80. 22683512 10.1016/j.exger.2012.05.021

[28] Fragala MS , Jajtner AR , Beyer KS , Townsend JR , Emerson NS , Scanlon TC , Oliveira LP , Hoffman JR , Stout JR . Biomarkers of muscle quality: N‐terminal propeptide of type III procollagen and C‐terminal agrin fragment responses to resistance exercise training in older adults.. J Cachexia Sarcopenia Muscle 2014;5:139–148. 24197815 10.1007/s13539-013-0120-z PMC4053565

[29] Rhee CM , Kalantar‐Zadeh K . Resistance exercise: an effective strategy to reverse muscle wasting in hemodialysis patients? J Cachexia Sarcopenia Muscle 2014;5:177–180. 25163460 10.1007/s13539-014-0160-z PMC4159495

[30] Kasner SE . Clinical interpretation and use of stroke scales. Lancet Neurol 2006;5:603–612. 16781990 10.1016/S1474-4422(06)70495-1

[31] Lincoln N , Leadbitter D . Assessment of motor function in stroke patients. Physiotherapy 1979;65:48–51. 441189

[32] Collen FM , Wade DT , Robb GF , Bradshaw CM . The Rivermead Mobility Index: a further development of the Rivermead Motor Assessment. Int Disabil Stud 1991;13:50–54. 1836787 10.3109/03790799109166684

[33] Kushner RF . Bioelectrical impedance analysis: a review of principles and applications. J Am Coll Nutr 1992;11:199–209. 1578098

[34] Selberg O , Selberg D . Norms and correlates of bioimpedance phase angle in healthy human subjects, hospitalized patients, and patients with liver cirrhosis. Eur J Appl Physiol 2002;86:509–516. 11944099 10.1007/s00421-001-0570-4

[35] Lukaski HC , Johnson PE , Bolonchuk WW , Lykken GI . Assessment of fat‐free mass using bioelectrical impedance measurements of the human body. Am J Clin Nutr 1985;41:810–817. 3984933 10.1093/ajcn/41.4.810

[36] http://www.neurotune.com/tl_files/neurotune/pdf/NTCAF_ELISA_Manual.pdf.

[37] Steubl D , Hettwer S , Vrijbloed W , Dahinden P , Wolf P , Luppa P , *et al* C‐terminal agrin fragment—a new fast biomarker for kidney function in renal transplant recipients. Am J Nephrol 2013;38:501–508. 24356308 10.1159/000356969

[38] Steubl D , Hettwer S , Dahinden P , Wolf P , Luppa P , Wagner CA , *et al* Influence of high‐flux hemodialysis and hemodiafiltration on serum C‐terminal agrin fragment levels in end‐stage renal disease patients. Transl Res 2014;164:392–399. 24907476 10.1016/j.trsl.2014.05.005

[39] Drey M , Behnes M , Kob R , Lepiorz D , Hettwer S , Bollheimer C , *et al* C‐Terminal Agrin Fragment (CAF) reflects renal function in patients suffering from severe sepsis or septic shock. Clin Lab 2015;61:69–76. 25807640 10.7754/clin.lab.2014.140724

[40] Ferns MJ , Campanelli JT , Hoch W , Scheller RH , Hall Z . The ability of agrin to cluster AChRs depends on alternative splicing and on cell surface proteoglycans. Neuron 1993;11:491–502. 8398142 10.1016/0896-6273(93)90153-i

[41] Bezakova G , Helm JP , Francolini M , Lømo T . Effects of purified recombinant neural and muscle agrin on skeletal muscle fibers in vivo. J Cell Biol 2001;153:1441–1452. 11425874 10.1083/jcb.153.7.1441 PMC2150725

[42] Bolliger MF , Zurlinden A , Lüscher D , Bütikofer L , Shakhova O , Francolini M , *et al*, Kunz B , Sonderegger P . Specific proteolytic cleavage of agrin regulates maturation of the neuromuscular junction. J Cell Sci 2010;123:3944–3955. 20980386 10.1242/jcs.072090

[43] Lieth E , Fallon JR . Muscle agrin: neural regulation and localization at nerve‐induced acetylcholine receptor clusters. J Neurosci 1993;13:2509–2514. 8388922 10.1523/JNEUROSCI.13-06-02509.1993 PMC6576499

[44] Fumagalli G , Balbi S , Cangiano A , Lømo T . Regulation of turnover and number of acetylcholine receptors at neuromuscular junctions. Neuron 1990;4:563–569. 2322461 10.1016/0896-6273(90)90114-u

[45] Rutkove SB , Aaron R , Shiffman CA . Localized bioimpedance analysis in the evaluation of neuromuscular disease. Muscle Nerve 2002;25:390–397. 11870716 10.1002/mus.10048

[46] Kafri MW , Potter JF , Myint PK . Multi‐frequency bioelectrical impedance analysis for assessing fat mass and fat‐free mass in stroke or transient ischaemic attack patients. Eur J Clin Nutr 2014;68:677–682. 24398644 10.1038/ejcn.2013.266

[47] Treviño‐Aguirre E , López‐Teros T , Gutiérrez‐Robledo L , Vandewoude M , Pérez‐Zepeda M . Availability and use of dual energy X‐ray absorptiometry (DXA) and bio‐impedance analysis (BIA) for the evaluation of sarcopenia by Belgian and Latin American geriatricians. J Cachexia Sarcopenia Muscle 2014;5:79–81. 24442632 10.1007/s13539-013-0126-6 PMC3953316

[48] Pateyjohns IR , Brinkworth GD , Buckley JD , Noakes M , Clifton PM . Comparison of three bioelectrical impedance methods with DXA in overweight and obese men. Obesity (Silver Spring) 2006;14:2064–2070. 17135624 10.1038/oby.2006.241

[49] Janssen I , Heymsfield SB , Baumgartner RN , Ross R . Estimation of skeletal muscle mass by bioelectrical impedance analysis. J Appl Physiol *(1985)* 2000;89:465–471. 10926627 10.1152/jappl.2000.89.2.465

[50] Heymsfield SB , Adamek M , Gonzalez MC , Jia G , Thomas DM . Assessing skeletal muscle mass: historical overview and state of the art. J Cachexia Sarcopenia Muscle 2014;5:9–18. 24532493 10.1007/s13539-014-0130-5 PMC3953319

[51] Berneis K , Keller U . Bioelectrical impedance analysis during acute changes of extracellular osmolality in man. Clin Nutr 2000;19:361–366. 11031076 10.1054/clnu.2000.0133

